# Advanced Diffusion MRI of the Visual System in Glaucoma: From Experimental Animal Models to Humans

**DOI:** 10.3390/biology11030454

**Published:** 2022-03-16

**Authors:** Monica Mendoza, Max Shotbolt, Muneeb A. Faiq, Carlos Parra, Kevin C. Chan

**Affiliations:** 1 Department of Biomedical Engineering, Tandon School of Engineering, New York University, New York, NY 11201, USA; mm8738@nyu.edu (M.M.); ms9785@nyu.edu (M.S.); 2 Department of Ophthalmology, NYU Grossman School of Medicine, NYU Langone Health, New York University, New York, NY 10017, USA; muneeb.faiq@nyulangone.org (M.A.F.); carlos.parragarzon@nyulangone.org (C.P.); 3 Department of Radiology, Neuroscience Institute, NYU Grossman School of Medicine, NYU Langone Health, New York University, New York, NY 10016, USA

**Keywords:** glaucoma, diffusion, magnetic resonance imaging, visual pathway, eye, optic nerve

## Abstract

**Simple Summary:**

This review summarizes current applications of advanced diffusion magnetic resonance imaging (MRI) throughout the glaucomatous visual system, focusing on the eye, optic nerve, optic tract, subcortical visual brain nuclei, optic radiations, and visual cortex. Glaucoma continues to be the leading cause of irreversible blindness worldwide and often remains undetected until later disease stages. The development of non-invasive methods for early detection of visual pathway integrity could pave the way for timely intervention and targeted treatment strategies. Principles of diffusion have been integrated with MRI protocols to produce a diffusion-weighted imaging modality for studying changes to tissue microstructures by quantifying the movement of water molecules in vivo. The development and applications of diffusion MRI in ophthalmology have allowed a better understanding of neural pathway changes in glaucoma. The feasibility of translating diffusion MRI techniques to assess both humans and experimental animal models of glaucoma and other optic neuropathies or neurodegenerative diseases is discussed. Recent research focuses on overcoming limitations in imaging quality, acquisition times, and biological interpretation suggest that diffusion MRI can provide an important tool for the non-invasive evaluation of glaucomatous changes in the visual system.

**Abstract:**

Glaucoma is a group of ophthalmologic conditions characterized by progressive retinal ganglion cell death, optic nerve degeneration, and irreversible vision loss. While intraocular pressure is the only clinically modifiable risk factor, glaucoma may continue to progress at controlled intraocular pressure, indicating other major factors in contributing to the disease mechanisms. Recent studies demonstrated the feasibility of advanced diffusion magnetic resonance imaging (dMRI) in visualizing the microstructural integrity of the visual system, opening new possibilities for non-invasive characterization of glaucomatous brain changes for guiding earlier and targeted intervention besides intraocular pressure lowering. In this review, we discuss dMRI methods currently used in visual system investigations, focusing on the eye, optic nerve, optic tract, subcortical visual brain nuclei, optic radiations, and visual cortex. We evaluate how conventional diffusion tensor imaging, higher-order diffusion kurtosis imaging, and other extended dMRI techniques can assess the neuronal and glial integrity of the visual system in both humans and experimental animal models of glaucoma, among other optic neuropathies or neurodegenerative diseases. We also compare the pros and cons of these methods against other imaging modalities. A growing body of dMRI research indicates that this modality holds promise in characterizing early glaucomatous changes in the visual system, determining the disease severity, and identifying potential neurotherapeutic targets, offering more options to slow glaucoma progression and to reduce the prevalence of this world’s leading cause of irreversible but preventable blindness.

## 1. Introduction

Glaucoma is one of the leading causes of irreversible blindness worldwide, with current estimates indicating 1 in 25 people aged 40–80 years old live with glaucoma [1]. Despite its prevalence, the etiology remains unclear, and there is currently no cure [2]. The term “glaucoma” refers to a group of heterogenous optic neuropathies, and primary open-angle glaucoma is the most predominant variant of these disease conditions [3]. Glaucoma is characterized by optic disk cupping and progressive, relatively selective retinal ganglion cell death that results in structural damage to the optic nerve and gradual reduction in the visual field [4,5]. Though intraocular pressure homeostasis is necessary to maintain eye shape and reduce the risk of mechanical strain on the optic nerve head [6], contrary to common belief, glaucoma may not necessarily be accompanied by high intraocular pressure. Normal-tension glaucoma is the medical term for the condition in this regard [7], accounting for an estimated 11% of glaucoma cases or just under one-third of open-angle glaucoma cases [8,9]. Likewise, patients with ocular hypertension could experience no progressive vision loss, indicating that there are other harmful factors or changes contributing to the degeneration of the optic nerve. Though the mechanisms by which intraocular pressure contributes to glaucoma progression are not completely understood, high intraocular pressure [10], along with age [11,12], genes [13,14], diabetes [15], myopia [16], ethnicity [17], and family history [5] are recognized as some of the driving risk factors for glaucoma onset. There is also evidence, albeit conflicting, suggesting environmental- and lifestyle-related factors that may pose a risk in the onset of the disease [6]. Some studies also reported altered levels of serum and aqueous humor trace elements such as zinc and iron in glaucoma patients [18].

Optic nerve damage and resulting vision loss are, as of now, irreversible and the probable multiple etiologies make glaucoma difficult to treat. The effects of glaucoma-related damage are further exacerbated by age-related nerve fiber loss, estimated at 4000–5426 nerve fibers per year in the non-diseased state [19]. Treatments that reduce elevated intraocular pressure are helpful for glaucoma management and improving patient quality of life, but they offer no direct neuroprotective mechanism to slow down or reverse retinal ganglion cell apoptosis to prevent eventual blindness [12]. Intraocular pressure is also a dynamic parameter that fluctuates, complicating the monitoring of these treatments, which are often unsuccessful [20]. Other risk factors are not easily targetable for intervention. Clinicians and researchers are working towards earlier detection of glaucomatous changes to begin treatment plans sooner to offer an improved outlook for decelerating complete vision loss and for reducing the impact of aging. Unfortunately, early intervention has proved challenging due to the heterogeneity of the disease with few widely accepted glaucomatous biological markers and non-invasive detection methods. People living with glaucoma are often asymptomatic until the condition has advanced to noticeable, irreversible neural damage that involves central vision loss, or they misattribute vision problems to other causes [5]. This makes glaucoma one of the most underdiagnosed ophthalmic conditions, a situation worsened by much of the world having varying access to eye care and the often long periods before patients receive a glaucoma diagnosis [21]. These are only a few of the issues that clinicians and researchers face during efforts to prevent the progression to complete blindness in glaucoma patients.

Non-invasive medical imaging offers the potential to resolve some gaps in our understanding of glaucomatous damage in the visual system. The microscopic nature of optic nerve damage presents a challenge, mainly with most conventional medical imaging modalities having resolutions on the order of millimeters while optic nerve axons average about a micrometer in diameter [19,22]. Significant strides were made in optical coherence tomography of the glaucomatous retina. Data derived from optical coherence tomography have displayed thinning of the peripapillary retinal nerve fiber layer (RNFL) and macular ganglion cell-inner plexiform layers consistent with glaucoma progression [23]. Through this evidence, glaucoma assessment commonly relies on the detection of RNFL changes, and other optic nerve head structural information [24]. However, shortcomings in these methods exist, such as test-retest reliability between patients, intrinsic floor effects for detecting progressive RNFL changes from advanced to end-stage glaucoma, the confounding effects of age or other vision conditions such as myopia and cataract, and limited penetration depth from optical imaging that hinders its utility for evaluating deeper brain structures [25]. When considering the hypothesis of glaucoma as a neurodegenerative disease of the visual system [26,27,28,29], few non-invasive imaging modalities allow for the detection of oxidative damage or changes in cellular processes such as transport behavior during early stages [30]. In later-stage glaucoma, significant changes to optic nerve size can be observed with conventional magnetic resonance imaging (MRI) or ultrasound biomicroscopy, but this offers little advantage for identifying the disease before the onset of progressive vision loss nor for direct identification of neurotherapeutic targets besides intraocular pressure reduction [31].

### Magnetic Resonance Imaging and Principles of Diffusion

**Figure 1:**
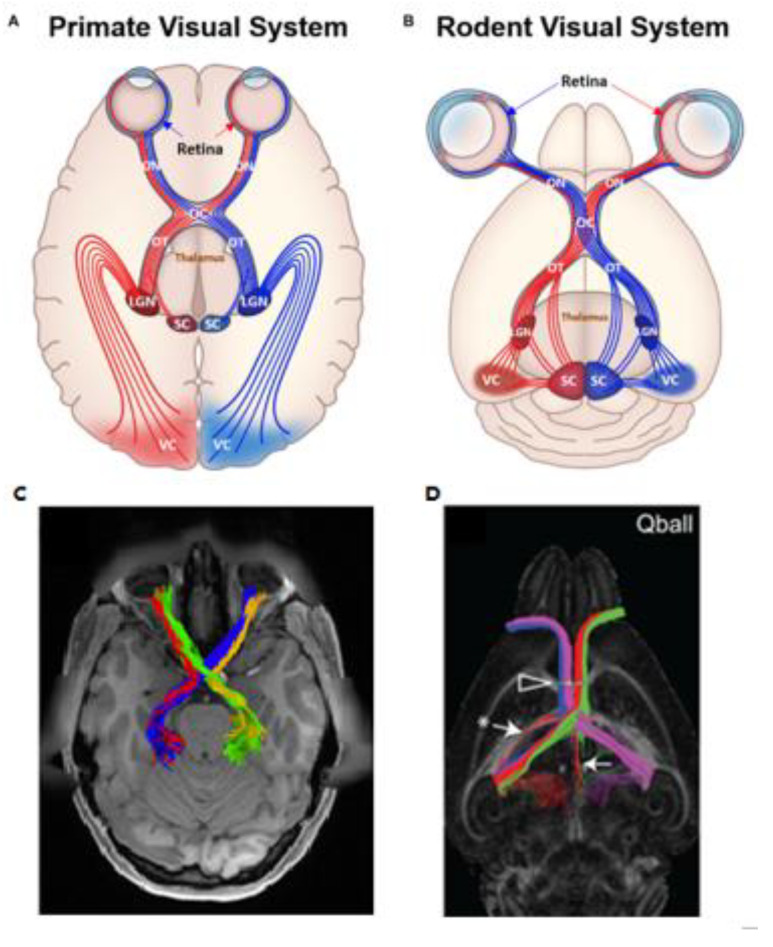
Representative illustrations (top) and diffusion MRI tractography (bottom) of primate (**A**,**C**) and rodent (**B**,**D**) visual systems. Visual structures including the retina, optic nerve (ON), optic chiasm (OC), optic tracts (OT), lateral geniculate nucleus (LGN), superior colliculus (SC), and visual cortex (VC) are labeled in the illustrations (top). Images are adapted with permission from Deng et al. [36]). Red and blue fiber tracts (**A**,**B**) represent proportion of projections from the left (L) and right (R) VC, respectively. Note the larger proportion of ON decussation in rodents than primates at the OC. Human optic nerve tractography (**C**) can depict both crossing (blue/green) and ipsilateral (red/yellow) projections. Image is adapted with permission from He et al. [37]. The mouse brain q-ball tractography (**D**) also demonstrates ipsilateral and contralateral projections at the optic nerve (white arrowhead), whereas white arrows and asterisk indicate areas where the model fitting fails. Image is adapted with permission from Moldrich et al. [38].

Researchers and ophthalmologists are turning to more advanced MRI techniques to image beyond the anatomical structure of the optic nerve and explore biomarkers of early glaucomatous states, potentially before the onset of detectable clinical changes [32]. MRI is a medical imaging modality that leverages strong magnetic fields to observe the proton behavior of water, abundant in biological tissue. Apart from human applications, it is also employed in preclinical animal research for studying the anatomy [33,34], function [35], and flow of physiological fluids such as blood and cerebrospinal fluid [34], among other applications in a non-invasive manner. Figure 1 offers a graphical depiction of the anatomical structures in the primate and rodent visual systems [36]. This similar organization facilitates both forward and reverse translations of MRI studies between glaucomatous animal models and humans and helps researchers make inferences for the disease conditions.

MRI is often preferred over other imaging modalities for brain assessments due to no-depth limitation, non-ionizing radiation, and the availability of various image scanning protocols that can result in a range of resolutions and specificity. For instance, functional MRI and structural MRI are effective in deciphering abnormalities in the interactions between the eye and brain’s visual system in disease states [32,39,40]. In high-tension glaucoma patients, functional MRI with visual stimulation showed reduced activation along the visual pathway as compared to normal-tension glaucoma patients [41]. On the other hand, structural MRI has been used for comparing lateral geniculate nucleus volumes between primary open-angle glaucoma patients and healthy controls [42], imaging the corpus callosum in infants with primary congenital glaucoma [39], investigating morphological changes in the glaucomatous visual cortex [43], and evaluating strain and straightness of the optic nerve in the presence and absence of elevated intraocular pressure [44], among other assessments of glaucomatous visual structures [40,45].

Despite prominent investigations of macro structures in the central nervous system of glaucoma patients, challenges remain when imaging structures on the order of microns, such as the optic nerve partly due to the trade-off between spatial and temporal resolution in MRI. Susceptibility to image artifacts is also increased by natural eye movements as well as partial volume effects from the surrounding cerebrospinal fluid and neighboring structures [19]. Rather than limiting imaging to macro structures, principles of diffusion and spatial localization can be leveraged to produce an MRI variant with diffusion-weighted signals stemming from quantification of the displacement of water molecules in an image voxel [46]. The diffusion of water molecules in the nervous tissue is heavily driven by thermal energy, otherwise known as Brownian motion. In a homogenous medium, the root mean square displacement of diffused molecules follows a Gaussian distribution where displacement in any direction has equal probability [47]. Diffusion-weighted images (DWI) can be generated with these principles where voxel signal intensity is proportional to the rate of diffusion across different tissues [48]. Using the diffusion MRI (dMRI) technique, estimation of diffusion gradients is achievable by recording the phase shift of the protons in biological tissues in response to applied consecutive spatial gradient pulses, incorporated in the design of the magnetic resonance pulse sequence. When a gradient is applied and then reversed with identical magnitude, protons that have remained in place do not display a phase shift, while a phase shift will be observed for protons that are displaced [49]. Therefore, the image contrast is indicative of the degree of restrictive diffusion along one direction within a voxel. In axons wrapped in the myelin sheath, diffusion is highly anisotropic, with the principal axis of diffusion described as the tensor [50]. The microscopic nature of proton-heavy water molecules is what permits increased sensitivity of the MR imaging modality to structures of varying permeabilities, such as myelin and neuronal organelles. This allows researchers to describe and identify resulting damage from neuronal diseases, including demyelination, synaptic pruning, and axonal loss [46]. 

The combination of glaucoma disease prevalence and global burden, its degenerative nature, and the challenges of detecting changes in optic nerve integrity and beyond triggered research into dMRI applications within the visual pathway of both human patients and glaucoma animal models. This review is centered on dMRI analysis of abnormalities of the eye and the visual pathway in glaucoma. Additionally, novel research methods employing dMRI variants for improving specificity for glaucoma interpretation are discussed. We expect that this review can better inform biologists, radiologists, and clinicians alike of the prospectives of dMRI for non-invasive and longitudinal understanding of glaucomatous neurodegeneration and neurotherapeutic options in both basic research and clinical translations.

## 2. Materials and Methods

### 2.1. Literature Search

This review documented dMRI applications, as well as associated methods applicable to the study of glaucoma from journal articles and conference proceedings. Sources were found using the PubMed database. Search results for journal articles that described glaucoma applications were generated by searching “glaucoma” coupled with the following search terms: “MRI”, “Structural MRI”, “Diffusion Tensor Imaging”, “Diffusion Kurtosis Imaging”, “Diffusion Weighted Imaging”, “diffusion MRI”, “white matter tract integrity”, and “tractography”. Data were excluded if research was not quantitative and did not provide a breakdown of cohort ages and MRI field strength. Data were also excluded if the focus of the research was not on either the optic nerve, optic tract, eye, subcortical visual brain nuclei, lateral geniculate nucleus, optic radiation, or the visual cortex. Journal articles that described methods potentially translatable to glaucoma were included if the same search query showed results for study on optic neuropathies other than glaucoma or on white matter neuropathies outside the visual system on a limited basis with respect to the values and relevance to prospective research designs in glaucoma study.

### 2.2. Anatomical MRI in Glaucona

**Figure 2:**
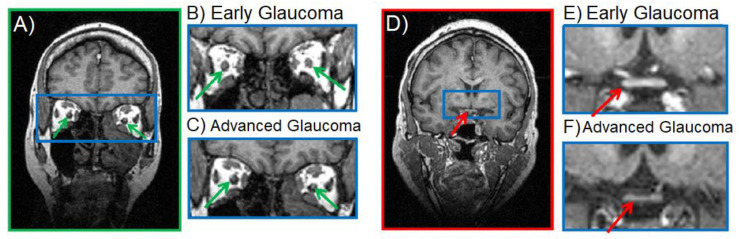
Anatomical MRI of the optic nerve (**A**, **green arrows**) and optic chiasm (**D**, **red arrows**) during early (**B**,**E**) and advanced stage (**C**,**F**) glaucoma. Image accreditation to Kasi et al. [51].

Conventional anatomical MRI can be useful for the detection of atrophy in the glaucomatous visual pathway, such as the progressive reduction in the human optic nerve and optic chiasm volumes toward advanced glaucoma stages [43,51] (Figure 2), and the age-dependent alterations to eye morphology in glaucoma animal models [52]. Beyond the anterior visual pathway [53], structural MRI also revealed reduced height and volume of the lateral geniculate nucleus and injury in the visual cortex of glaucoma patients with mild to severe visual field defects [43,54,55,56] with changes to the cortical thickness and regional volumes that correlate with disease severity [40,57]. These results are consistent with histological observations in patients or primate models of glaucoma [58].

While elevated intraocular pressure could result in mechanical damage to the optic nerve, mechanical damage could also occur from other phenomena. Demer et al. used structural MRI to image the optic nerve in the presence of pulling, tractional forces [44]. Quantified by computing Cartesian distances to determine the ratio between mid-orbital lengths and minimum optic nerve path lengths, optic nerve straightness was compared between normal-tension glaucoma patients and healthy controls. Adduction conditions resulted in increased retraction of the globe of the glaucomatous eye as compared to controls, suggesting that mechanical stress on the optic nerve independent of intraocular pressure is present in glaucomatous states.

The macroscopic changes detectable by structural MRI are often observed near advanced stages of glaucoma when vision loss is already underway and neural damage is evident. Conversely, diffusion weighting can assist in detecting microstructural changes as these methods appear to be sensitive to inflammation [59], demyelination [60], and axonal loss or injury [61] during various stages of the disease. In the following sections, we briefly describe the biophysics of DWI, diffusion tensor imaging (DTI), and higher-order dMRI methods followed by how sensitive and specific these dMRI techniques can be used for non-invasive evaluation of ocular and brain microstructures in glaucoma.

### 2.3. Diffusion-Weighted Imaging (DWI) in Glaucoma

Diffusion-weighted images are acquired at varying diffusion gradient strengths and timing factors that are termed diffusion weighting factors or *b*-values. Apparent diffusion maps, made from normalized diffusion values, are generated by acquiring data from multiple *b*-values during MRI scanning to quantify the diffusion weighting along one diffusion direction. The apparent diffusion coefficient (ADC) denoted *D* in mm^2^/s is calculated with the Stejskal–Tanner equation. The ADC simplifies the average trace (average sum of the eigenvalues) of the diffusion tensors at every voxel. The tensors are obtained by estimating from the ADC taken in different directions [62]. The relationship between the MR signal intensity in the DWI scan (*S*), the *b*-value, and the ADC is summarized by Equation (1), where *S_0_* is the baseline signal intensity without diffusion weighting, i.e., at b = 0 mm^2^/s (b_0_) [48]:

*S* = *S_0_*·e^(−*bD*)^ (1)

Within the eyeball, DWI was used to observe age-dependent changes in mean ADC in the vitreous of healthy human subjects from toddlers to over 71 years old [63]. The researchers attributed this to the suspected age-driven breakdown of collagen fibrils in the vitreous humor resulting in changes to cell organization and microcirculation. For the retina, DWI can provide better contrast than non-DWI b_0_ images in differentiating loss of integrity in a mouse model of retinal degeneration (*rd1)* versus wild-type mice [64]. Glaucoma induced via chronic ocular hypertension was shown to result in the loss of cone photoreceptors [65], apart from retinal ganglion cells. This suggests DWI may possess potential in monitoring layer-specific retinal changes under different glaucomatous conditions.

In the brain’s visual system, with the assumption of homogeneity in every diffusion direction, DWI on its own is more commonly applied in grey matter analysis when average diffusion values per voxel are sufficient since the grey matter is relatively isotropic [66]. White matter tracts are anisotropic and require further mathematical processing to derive meaningful measures on structure and complexity. There are currently a number of DWI analytic models whose data can be collected within a clinically feasible timeframe, such as diffusion tensor imaging (DTI), diffusion kurtosis imaging (DKI), and white matter tract integrity (WMTI) models [67]. WMTI is an extension of DKI, providing the biophysical explanation of the white matter microstructure to accompany the values outputted by DKI [62].

### 2.4. Diffusion Tensor Imaging (DTI) in Glaucoma

Diffusion tensor imaging (DTI) is a technique that uses diffusion-weighted scans acquired in different directions to provide microstructural information based on measures of anisotropy and restricted water diffusion within physiological structures [68,69]. The anisotropic nature of biological tissues results in diffusion varying in any direction, which can be represented simplistically by a 3 × 3 array tensor calculated from the ADC approximation. The eigenvalues (*λ_i_*) of the diffusion tensor can be used to describe the magnitude of the diffusion and level of directionality, whereas the eigenvectors can be used to describe the orientation. Ultimately, the DTI model can derive rotationally invariant biophysical parametric maps of fractional anisotropy (FA), axial diffusivity (AD), radial diffusivity (RD), and mean diffusivity (MD). FA represents the variance of the eigenvalues and is a normalized value that ranges from 0 to 1, where 0 indicates isotropy [50]. AD describes the diffusion in the principal direction, and diffusion perpendicular to AD is described by RD. MD describes the magnitude of the diffusion overall [47,70]. Table 1 summarizes how each DTI parameter is calculated from the eigenvalues.

**Figure 3:**
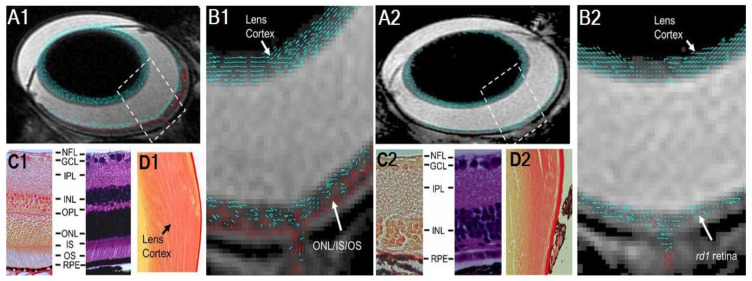
Reduction in fractional anisotropy in the outer retina layers of *rd1* mice (**A2**,**B2**) relative to wild-type controls (**A1**,**B1**) (red vectors), consistent with histological evidence of photoreceptor layer deterioration. Images are adapted with permission from Chen et al. [64].

DTI measurements of water diffusion through structures that traverse a single axis in a highly restrictive manner would expectedly result in high FA values, indicative of strong directionality. Because of this, in white matter tissues, FA is sensitive to overall microstructural integrity [71], AD provides an indication of axonal integrity [72], and RD can help infer integrity of the myelin sheath among other factors [47,70]. Within the eye, DTI also allows layer-specific assessments of fibrous structures. For instance, lowered anisotropy was observed through DTI together with a lack of perpendicular directionality in the outer retina of the *rd1* mouse model of retinal degeneration, which was subsequently confirmed via histology, indicative of photoreceptor cell damage (Figure 3) [64].

**Figure 4:**
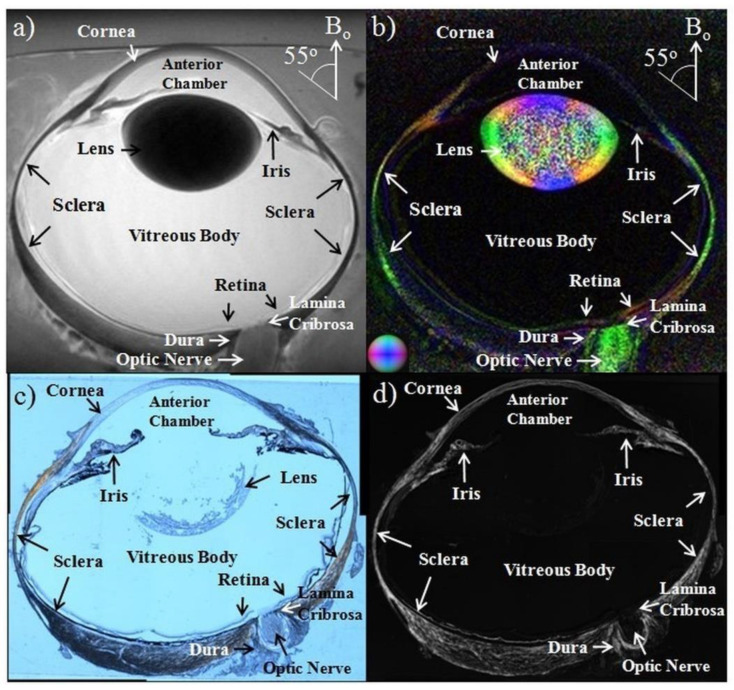
Whole ovine eye images acquired with T2-weighted MRI (**a**), color-encoded DTI mapping for fractional anisotropy (**b**), polarized light microscopy of a histological section (**c**), and intensity mapping of collagen density (**d**). Colors in (**b**) correspond to principal diffusional directions: caudal-rostral (blue), dorsal-ventral (green) and left-right (red). Images are adapted with permission from Ho et al. [73].

DTI methods were also applied to examine the corneoscleral shell of the eyeball (Figure 4) [73], with its anisotropy and diffusion orientations confirmed by imaging the fiber arrangements histologically. Here, the effects of intraocular pressure loading may increase the FA of the collagen fibers in the sclera and cornea, whereas treatments with increasing glyceraldehyde (mimicking crosslinking conditions) and chondroitinase ABC concentrations (mimicking glycosaminoglycan depletion) decreased diffusivities. The microstructural organization and composition of the corneoscleral shell determine the biomechanical behavior of the eye and are important in diseases such as glaucoma and myopia. DTI can combine with other MRI modalities to help evaluate the pathophysiological mechanisms in the corneoscleral shell and the efficacy of corneoscleral treatments.

**Figure 5:**
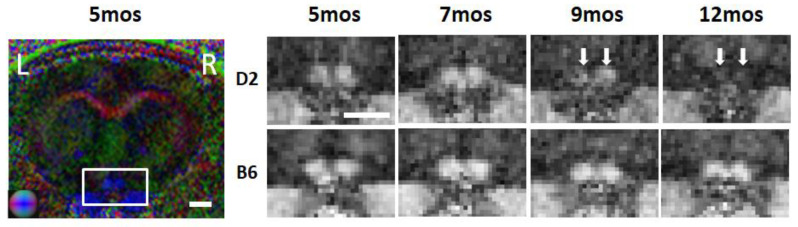
Longitudinal DTI at the level of the optic nerve (white rectangle) in the DBA/2J experimental glaucoma mouse model (D2) and healthy C57BL/6J mice (B6). (**Left**) Color-coded fractional anisotropy (FA) directionality map along caudal-rostral (blue), left-right (red), and dorsal–ventral (green) directions in a D2 mouse at 5 months old (mos). (**Right**) FA value maps of the D2 and B6 optic nerves from 5 to 12 mos. White arrows point to the deteriorating D2 optic nerves at 9 and 12 mos when intraocular pressure increased alongside the same period. Image adapted with permission from Yang et al. [12].

DTI has been increasingly used to evaluate glaucomatous damage in both rodent models [12,73] and patients with and without visual field defects [31,74,75] (Table 2), showing FA and directional diffusivities as robust and sensitive DTI parameters of disrupted structural integrity across species. In experimental animal models, Yang et al. used DTI for examination of age-related changes in the visual pathway integrity of DBA/2J mice from 5 to 12 months old [12], showing altered FA and RD of the optic nerve (Figure 5) and optic tract along with elevated intraocular pressure beginning at 8–9 months old. Results from a DTI study using 5 different induced or genetic animal models of glaucoma exhibited FA, RD, and AD changes along the visual pathway at varying rates, which may reflect different severity and timing of glaucomatous damage [76].

**Figure 6:**
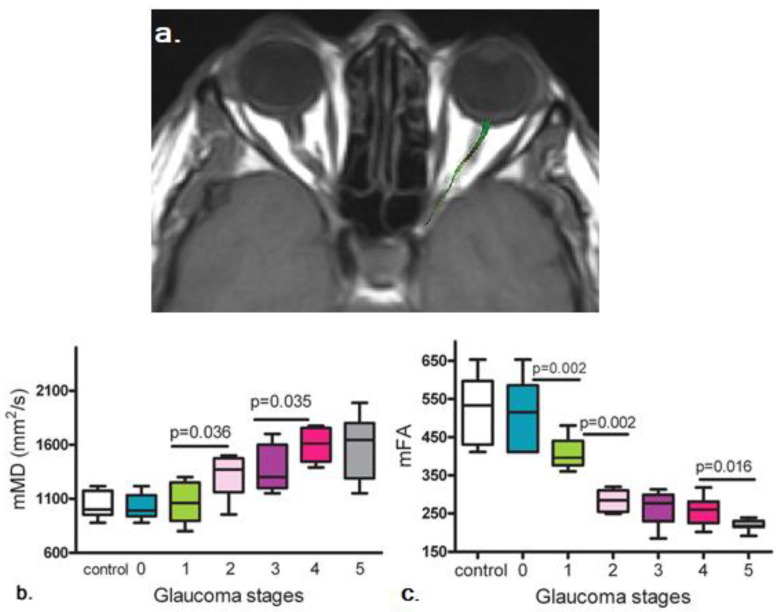
Changes in DTI parameters at different glaucoma stages. (**a**) Representative anatomical T1-weighted image from a 65-year-old male with severe glaucoma in the left eye (right side of image), with the optic nerve delineated by the green region of interest. Glaucoma progression is characterized by an increase in mean diffusivity (**b**) and by a decrease in fractional anisotropy (**c**) in the optic nerve. Glaucoma subjects were staged according to their visual field mean deviation score (MDS) as (0) increased intraocular pressure without visual field defects, MDS greater than 0 dB; (1) early, with an MDS between −0.01 and −6.00 dB; (2) moderate, with an MDS between −6.01 and −12.00 dB; (3) advanced, with MDS between −12.01 and −20.00 dB, (4) severe, with an MDS greater than −20.01 dB; (5) end-stage glaucoma. Images are reproduced from Garaci et al. [75].

In humans, an early DTI study combined tractography and region-of-interest analysis to demonstrate lower FA and higher MD in the glaucomatous optic nerve [75]. Figure 6 illustrates the progression of mean MD and mean FA changes at increasing stages of glaucoma. DTI also revealed damaged anatomical connectivity in primary open-angle glaucoma patients detectable in the early stages of the disease within and beyond the visual system [77,78]. These DTI observations added to the wealth of evidence indicating the neurodegenerative nature of glaucoma [27], with widespread involvements throughout the brain [77].

The lower FA in the glaucomatous optic nerve could be correlated with lower RNFL thickness [79,80]. As summarized in Table 2, the lower FA and higher RD and MD were consistently reported in the glaucomatous optic nerve in both normal-tension glaucoma and primary open-angle glaucoma [27,81], which could indicate the sensitivity of DTI to axonal disruption and myelin loss [72]. While Table 2 compares DTI metrics between glaucoma patients and healthy controls, similar changes were observed when comparing FA, MD, and RD changes between early and late-stage glaucoma [82].

There were contradictions observed in AD changes in glaucoma (Table 2), suggesting the metric may present differently in subjects across disease stages or with varying comorbid conditions or risk factors. For example, lower AD in the optic nerve was observed during early-stage glaucoma, which becomes higher again in late-stage glaucoma [83], suggestive of underlying glial activity or other repair mechanisms [84,85]. Adding more contention to reported DTI results, changes in FA and AD in the optic nerve may also depend on head tilt conditions, suggesting a strong role for cerebrospinal fluid volume and hydrodynamics in the observed diffusion activity [86].

**Figure 7:**
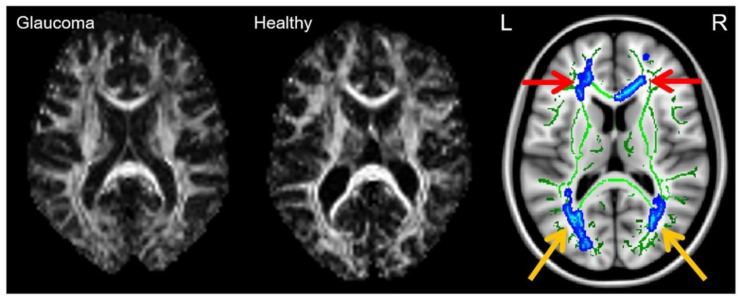
Fractional anisotropy (FA) maps at the level of the optic radiations in glaucoma (**left**) and healthy control subjects (**middle**). (**Right**) Colored maps of group comparisons, estimated with tract-based spatial statistics (TBSS), are overlaid on a standard MNI152 T1 MRI template. Green pixels correspond to the white matter tracts skeleton, and blue pixels correspond to brain regions of reduced FA in advanced glaucoma compared to early glaucoma at the optic radiations (yellow arrows) and frontal lobe white matter (red arrows). Images are reproduced from Murphy et al. [94].

Apart from the optic nerve, DTI is well-positioned to study deeper brain regions (Table 3) such as the optic radiations for the detection of trans-neuronal degeneration in glaucoma (Figure 7) due to the absence of depth limitation in MRI. However, it remains unclear if the earliest glaucomatous changes occur in the optic nerve, optic radiation, or elsewhere, and whether axonal and glial injuries occur simultaneously or sequentially in glaucoma [93]. More longitudinal studies, beginning with participants with a family history of glaucoma, would be beneficial in this regard.

### 2.5. Diffusion Kurtosis Imaging (DKI) in Glaucoma

Despite being sensitive for detecting glaucomatous changes in the visual pathway, DTI parameters present challenges in the interpretation of the underlying pathologies due to the limited specificity [47]. DTI is currently considered a conventional dMRI protocol for neuroscience and neurological research, but the simplicity of the single-compartment model and limitations such as the inability to resolve fiber crossings [62] present a need for further development. In addition, DTI assumes free water distribution with diffusion values following a normal (Gaussian) distribution [95]. This would misrepresent the stochastic biological systems with restrictive diffusion, rendering DTI an inaccurate model of brain integrity assessments [96]. DKI offers a solution to move towards biologically relevant explanations for DTI results by incorporating non-Gaussian water diffusion analyses. DKI estimates the kurtosis, or the 4th central moment of a distribution, of the probability distribution for water diffusion displacement in biological structures. In probability theory, the higher the kurtosis value, the sharper the peak of the probability distribution, and the more restrictive the water diffusion displacement is assumed [95].

Another way to conceptualize the differences between DTI and DKI models is to consider that DTI assumes homogenous water diffusion, whereas, in true biological systems, diffusion is highly heterogeneous in varying directions. DKI measures the deviation from the Gaussian distribution akin to homogenous diffusion, and this calculation can be observed in Equation (6), where *K_app_* is the apparent kurtosis value [95]. For these reasons, DKI is considered to be complementary to DTI for investigating white matter structure abnormalities as it does not restrict itself to the Gaussian model, making it a more specific method for drawing conclusions in biological structures with varying structural complexity [62]. Higher kurtosis values are associated with increased structural complexity since the values imply more interactions between water molecules and the surrounding cell membranes [95]. By contrast, as *K_app_* approaches zero, the closer the DKI model resembles the DTI model, and this can also be seen in (6). The three main DKI parametric outputs include mean kurtosis (MK), radial kurtosis (RK), and axial kurtosis (AK), which are kurtosis analogs inversely proportional to the tensor parameters in DTI [62].

ln(*S*(*b*)/S(0)) = −*bD* + 1/6 *b*^2^
*D*^2^ *K_app_* (6)

With an increased number of gradient directions and higher *b*-values for image acquisition over conventional DTI, kurtosis parameters offer higher specificity and may potentially serve as more reliable biomarkers for characterizing early glaucomatous damage in both white matter and gray matter [80]. DKI measurements are relatively new to ophthalmologic research. To date, studies have pointed to evidence of lower MK along the visual pathway [91] and lower RK along the optic tract in primary open-angle glaucoma patients [80]. Xu et al. reported significantly lower MK at the lateral geniculate nucleus in glaucoma patients with reported visual defects, which suggests neuronal loss [55] and decreased metabolic activity [97] reported in other studies. Apart from region-of-interest analysis, Nucci et al. used voxel-wise analysis to evaluate group differences in normal-appearing white matter in and outside the visual system [98]. Their results demonstrated significantly lower MK and RK in glaucoma patients compared to controls in the inferior longitudinal fasciculus, which connects the temporal and occipital lobes. The widespread changes observed outside the primary visual pathway could help explain the impairments in cognitive function some glaucoma patients experienced as well as disruption to higher-order neuronal pathways [99]. In normal-tension glaucoma patients, Li et al. reported a reorganization of information flow in the visual cortex based on significantly lower FA, RK, AK, and MK measurements compared to normal controls [100]. The lower RK and AK values were interpreted as decreased compactness of the myelin and axon as well as impaired overall microstructural integrity.

### 2.6. White Matter Tract Integrity (WMTI) Model in Glaucoma

Both DTI and DKI are based on diffusion value transformations and offer little information regarding the (patho-)physiological processes in different structural compartments [62]. A biophysical explanation of the white matter is necessary to obtain a more complete interpretation of the properties being described by DTI and DKI. The white matter tract integrity (WMTI) model is a two-compartment framework that describes the white matter tissue as a combination of the intra- and extra-axonal compartments. In this decomposition, the extra-axonal compartment is considered to have hindered or obstructed diffusion, and the intra-axonal compartment is considered to have restricted diffusion. In this context, restricted diffusion is considered non-Gaussian with minimal orthogonal diffusivity, while hindered diffusion would imply the opposite behavior [101].

Measures obtained using the WMTI model include tensors for the intra-axonal space (IAS) and extra-axonal space (EAS), the axonal water fraction (AWF), and the tortuosity of the extra-axonal space (*α_EAS_*) [62]. AWF is the proportion of water detected by the MRI acquisition in the axon as compared to the total water detected [46]. In order to implement this two-compartment approach, some assumptions must be put in place, specifically that the intra-axonal space should be modeled as an impermeable cylinder, with some degree of myelination, and the extra-axonal space should be modeled as a medium. Taken together, the mathematical model for the DWI signal intensity for each direction *n* becomes (7) [62]:

(*S*(*b*))/(*S*(0)) = *f*exp(−*bn^T^ D_IAS_* *n*) + (1 − *f*)exp(−*bn^T^ D_EAS_*
*n*) (7)

Here, *f* is the axonal water fraction, and *D* is each respective compartmental diffusion tensor. For any direction *i*, *D_IAS_* can be estimated by (8), and *D_EAS_* can be estimated by (9). WMTI follows the DTI model using DKI parameters, resulting in the eigenvalues of the matrices that represent the tensors corresponding to estimated physiological measurements [62].

*D_IAS_* = *D_i_* [1 + √((*K_i_* f)/(3(1 − f)))] (8)

*D_EAS_* = *D_i_* [1 − √((*K_i_*(1 − f)/(3f))] (9)

The primary eigenvalue of *D_EAS_*, *λ*_1,*EAS*_, corresponds to the axial EAS diffusivity. The average of eigenvalues *λ*_2,*EAS*_ and *λ*_3,*EAS*_ corresponds to the radial EAS diffusivity. The ratio between the axial EAS diffusivity and the radial EAS diffusivity corresponds to the tortuosity, denoted *α_EAS_*. *α_EAS_* can also be thought of as the ratio between the path or curve length and the total displacement. The trace of the *D_IAS_* tensor, the sum of all eigenvalues, corresponds to the intra-axonal diffusivity. At each voxel, the AWF, *f*, can be fundamentally estimated by (10), where *K*_max_ is the maximal kurtosis calculated from the diffusion in every direction [62].

*f_Kmax_* = *K_max_*/(*K_max_* + 3) (10)

**Figure 8:**
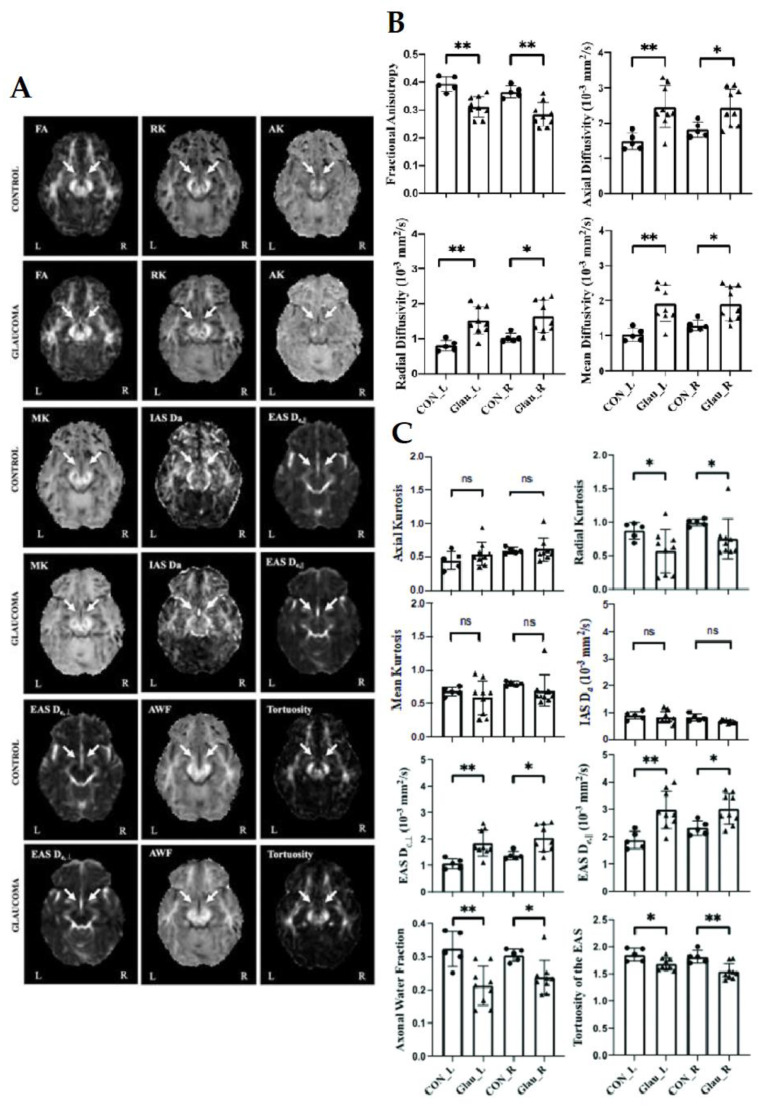
(**A**) Representative parametric maps of DTI [fractional anisotropy (FA)], DKI [radial kurtosis (RK), axial kurtosis (AK), mean kurtosis (MK)], and WMTI models [axial IAS diffusivity (D_a_), axial EAS diffusivity (D_e,//_), radial EAS diffusivity (D_e,__⊥_), axonal water fraction (AWF), tortuosity of the EAS (ratio of D_e,//_ and D_e,__⊥_)] from glaucoma and healthy control groups at the level of the optic tracts (arrows). (**B**,**C**) Quantitative comparisons of DTI (**B**) and DKI and WMTI parameters (**C**) in the left (L) and right (R) optic tracts of glaucoma (Glau) and healthy control (CON) groups. Unpaired t-tests between glaucoma and healthy groups, * *p* < 0.05; ** *p* < 0.01; ns: not significant. Images are adapted with permission from Sun et al. [80].

WMTI was coupled with DKI and DTI measurements for investigating the effects of aging [102], traumatic brain injury [103], and other neurodegenerative conditions [104] on the microstructural integrity of white matter. It is expected that the combination of these techniques could be leveraged to explain deviations from normal visual system characteristics in glaucoma. For example, an initial WMTI study by Sun et al. observed higher radial EAS diffusivity, higher axial EAS diffusivity, and lower AWF in the optic tract of glaucoma patients [80] (Figure 8A,C). These findings may also help explain the controversial AD results in the glaucoma DTI literature in terms of varying degrees of axonal loss, glial activity, or a combination thereof. The research group also reported dMRI correlates with clinical ophthalmic scores. Despite significant differences between glaucoma and healthy control groups for all DTI parameters (Figure 8B), no apparent correlation was found between DTI measurements and clinical severity in terms of ganglion cell-inner plexiform layer thickness, cup-to-disc ratio, or visual field mean deviation. Conversely, RK in DKI positively correlated with ganglion cell-inner plexiform layer thickness in the right optic tract of glaucoma patients. These observations could suggest higher sensitivity of advanced dMRI techniques such as DKI to glaucoma severity as compared to conventional DTI.

### 2.7. Diffusion MRI Tractography in Glaucoma

dMRI can be used to create reconstructions of fiber tracts along the visual pathway. Tractography methods for visualizing nerve fiber tracts and architecture using dMRI data allow for a non-invasive means for tracking orientation and directionality of white matter across the central nervous system in 3D space and may provide greater accuracy in the region-of-interest analysis [75]. Tract reconstructions may also allow for a better spatial understanding of the relationships between cortical regions and pathways. As essentially an extrapolation of voxel-wise dMRI estimates, diffusion tractography is based on the assumption that when diffusion is measured in varying directions, it will tend towards a “preferred” direction of diffusion that corresponds to the direction of the axon in healthy organisms or the direction in which the diffusion is the least obstructed or disrupted. Diffusion behavior is then quantified by directionally color-encoding maps based on fractional anisotropy, with retained principal vectors for diffusion tensors with high FA values and a different color for each plane. Tractography methods include probabilistic or deterministic algorithms, as well as multi-fiber or single-fiber models. In deterministic tractography, the vector field in each region of interest, also referred to as the target volume, is reconstructed from a seed voxel by generating tracks based on the best estimates of nerve fiber orientation. One limitation of deterministic methods is the lack of information regarding random or systematic errors. Without a margin of error, it is difficult to determine confidence in the results. Alternatively, probabilistic algorithms consider degrees of uncertainty. As the confidence decreases when the tract tracking is further from the seed volume, the probability distribution functions for fiber orientation are better defined at the central region around the starting point in probabilistic tractography. Besides limitations with regards to distal regions, probabilistic tractography is more computationally demanding than deterministic methods as generating probability distribution functions is an iterative process. Both methods are also limited in their ability to resolve fiber crossings [105].

You et al. suggested that probabilistic dMRI tractography could be ideal for modeling trans-synaptic neurodegeneration along the visual pathway [106]. Using this method, observations conducted on a cohort of primary open-angle glaucoma patients with binocular visual hemifield loss and another cohort of patients with optic neuritis suggested consistent demyelination of optic radiation fiber tracts preceding axonal loss during neurodegenerative spread. Researchers previously demonstrated these changes in rodent models of optic nerve injuries, and the results were also supported with multifocal visual evoked potential recordings. Hanekamp et al. used dMRI tractography to compare white matter tracts between glaucoma patients and patients with monocular blindness. Their results supported evidence for reorganization in the visual pathway of those with monocular blindness, whereas this compensatory mechanism appears absent or limited in glaucoma patients [107]. Though tractography demonstrated substantial structural changes at the early visual system, cautions should be taken due to the methodology limitations including the incidence of false tracts and decreased confidence away from the seed region [105]. Further proof of concept and investigations are necessary to help validate tractography methods for identifying irregularities, lesions, or disruptions along the visual pathway. Overall, advanced tractography and dMRI methods show promise for non-invasive replication of neuroanatomy and localization and for improving specific, targeted interventions for optic neuropathies.

## 3. Challenges and Limitations

### 3.1. Limitations of the Literature Review Methods

Recent dMRI of experimental animal models of glaucoma may represent certain aspects of glaucomatous optic neuropathy. However, to date, there is no single animal model that can fully represent primary open-angle glaucoma, normal-tension glaucoma, or other glaucoma types. Thus, the forward and reverse translations between dMRI of humans and experimental animal models of glaucoma should be interpreted with caution. Future studies may take the recent consensus recommendation for experimental glaucoma models into consideration when devising appropriate research questions relevant to glaucoma [108]. On the other hand, we did not discuss the effects of important risk factors such as ethnicity or age on human glaucoma dMRI observations, given the lack of information or small samples [17]. As the glaucoma dMRI literature continues to grow, a meta-analysis may be performed to account for multiple factors in study designs, including the glaucoma model used, magnetic field strength, age, ethnicity, bias risk, and confounding variables or conditions.

### 3.2. Limited Specificity from DWI and DTI Results

The main overarching pitfall of dMRI results is the over-extraction of information for biophysical and pathophysiological interpretations. The dMRI methods described whether mathematical or compartmental systems all represent a simplification of a stochastic biological system, which could lead to overfitting of results [109]. Though conventional DTI is widely applied in clinical and preclinical research, its limited specificity leads to several problems. For example, while ADC and MD are more commonly used in the clinic, a clear consensus for how to interpret their changes is currently lacking in the literature, within and outside ophthalmologic research. In a preclinical DTI study of the optic nerve, following retinal ischemia, the initial decrease to AD that preceded the increase to RD resulted in an initial decrease to MD at the time of axonal injury, coupled with the AD decrease. As myelin injury followed and RD increased, MD was offset and approached a net zero change [47]. Given the apparent normalization in MD in the presence of AD and RD changes, it is expected that examining directional diffusivities may help identify brain abnormalities more accurately than using MD or ADC alone. There is also an ongoing debate on the effects of anesthesia or hypercapnia on the dMRI quantitation [50,110]. Last but not least, though dMRI offers an opportunity to investigate changes in the visual pathway in vivo, progressive damage occurring in the presence or absence of elevated intraocular pressure, as well as in varying rates of loss of visual function, points to several disease mechanisms in glaucoma, necessitating more comprehensive measurements of the visual system. Despite the sensitive FA decrease in most dMRI studies indicative of loss of overall microstructural integrity along the glaucomatous visual pathway, with the inconsistency of mean and directional diffusivity changes across glaucoma studies, it is recommended to support DWI or DTI findings with higher-order dMRI models, including DKI, WMTI, or diffusion basis spectrum imaging, as well as other measurements such as optical coherence tomography, electroretinography, and histological validation in basic research [64] to determine more clearly the eye-brain interactions and pathophysiological events under different glaucoma conditions for better clinical translations [109]. 

### 3.3. Imaging Quality

Robust post-processing imaging protocols such as optimized motion correction and registration methods are necessary to facilitate image analysis of the visual system [111], especially for analysis of voxel-wise differences. However, the limited spatial resolution in dMRI data renders intrinsic challenges for the depiction of small but complex structures [112]. Moreover, while echo planar imaging sequence is currently the most common dMRI acquisition method of choice, it is prone to distortion artifacts where bone meets soft tissue and at the air-tissue interface. Multi-shot acquisition and parallel imaging in the form of multiband radiofrequency excitation are recent developments that help diminish these limitations. These novel methods may serve to improve the quality of imaging of orbital and retinal structures, including but not limited to dMRI [113].

Another limitation of dMRI is the inability of DTI to resolve fiber crossings, which could result in the emergence of inaccuracy in visual pathway tractography and false detection of FA changes in the optic chiasm [62]. The problem can become more compounded in glaucoma as optic nerve atrophy progresses and the surrounding cerebrospinal fluid moves into the atrophied space, creating confounding dMRI signals in the region of interest. *q*-space imaging (QSI), also known as diffusion spectrum imaging (DSI), shows promise in resolving intravoxel fiber crossing that DTI cannot at the expense of larger field gradients and time-intensive sampling requirements [114]. QSI measures the diffusion function directly in a three-dimensional lattice. As with DKI, the probability density function is modeled in a non-Gaussian manner, increasing biological relevance [115].

Due to its lengthy acquisition time, QSI is predominantly used in small organisms and non-biological applications. Novel QSI, namely q-ball imaging, resolves some of the sampling burden of QSI through the calculation of an orientation distribution function (ODF) rather than a single diffusion tensor from high-angular resolution diffusion imaging (HARDI) [114]. High-definition fiber tractography (HDFT) uses more advanced processing and reconstruction methods, including q-ball imaging, in order to map white matter tracts to cortical targets in the presence of fiber crossings and complex angulations, with little loss in on anatomical features such as gyrification patterns and the angulation of the Meyers loop in the optic radiations [116]. There are some contentious assumptions that accompany the method [117], making q-ball imaging still considered experimental before robust implementation for observing glaucomatous changes in the brain.

### 3.4. Reproducibility

It is challenging to compare dMRI results between studies due to the reproducibility from numerous confounding conditions in patients with glaucomatous abnormalities. In addition, currently, no standard DTI protocol exists, evident by the various acquisition parameters across studies. Different *b*-values, for example, may result in varying DTI parametric measures in the same brains [118]. dMRI can be an effective diagnostic tool when consistent results are achieved and receiver operating characteristic (ROC) curves evaluated with high area under curve (AUC) using comparable acquisition protocols for scans of patients with varying medical histories. Nevertheless, researchers in this field remain optimistic about the rate of new technology developments for improving the sensitivity and specificity of dMRI to the nervous tissues of the visual system, whether through acquisition or post-processing [113].

## 4. Future Prospective and Opportunities

### 4.1. Exploring Comorbid Conditions

**Figure 9:**
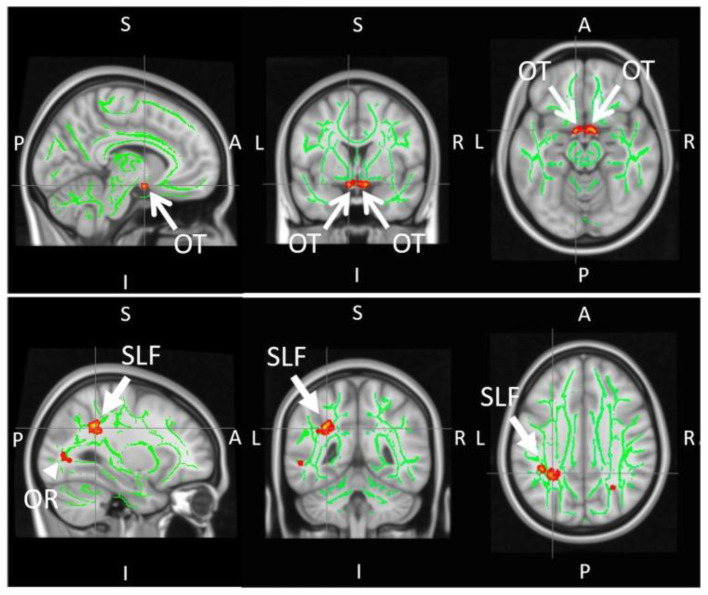
Tract-based spatial statistics (TBSS) of major white matter skeletons (green) demonstrating reduced FA (red and yellow pixels) in the optic tract (OT), optic radiation (OR), and the left superior longitudinal fasciculus (SLF) of early glaucoma patients as compared to healthy controls. Images are adapted with permission from Trivedi et al. [119].

There is growing evidence to suggest that glaucoma involves disruption to brain networks beyond the visual system. Visuomotor and postural coordination were shown to be altered in the glaucomatous state, resulting in falls and injuries beyond healthy aging [119]. A DTI study conducted by Trivedi et al. sought to investigate the neural underpinnings of postural control in glaucoma [119]. Tract-based spatial statistics (TBSS) demonstrated that early glaucoma patients had lower FA in the superior longitudinal fasciculus around the supramarginal gyrus, a key area for multisensory integration along with significantly smaller optic nerves compared to controls (Figure 9). These observations were consistent with task-free functional MRI results, which showed decreased functional connectivity between the supramarginal gyrus and visual occipital area, as well as the supramarginal gyrus and superior sensorimotor area in the glaucoma group.

Though not necessarily a neurodegenerative disease, there is dMRI evidence to suggest astronauts experience changes to white matter microstructure in zero-gravity environments, resulting in impaired balance post-space flight [120]. Gait and balance were also studied with dMRI in Parkinson’s patients, with DTI and DKI analyses conducted in motor control regions including the basal ganglia, putamen, thalamus, pons, and midbrain [121,122]. The parallels between the neuropathologies of glaucoma with other vision- and balance-related conditions support the translation of dMRI techniques and applications between different types of white matter injuries.

Another potential comorbidity that warrants further investigation is the incidence of sleep disorders in glaucoma patients. Vision loss can disrupt inputs to the suprachiasmatic nucleus, thereby impacting circadian rhythms [123]. The underlying mechanisms of this disruption are only just starting to be explored, but this comorbidity presents a prospective for further research into circadian rhythm disruption in glaucoma patients and experimental animal models. Recent work conducted by Bang et al. using functional MRI and metabolic magnetic resonance spectroscopy found significantly enhanced functional connectivity in the arousal system and decreased functional connectivity in sleep-promoting areas of the glaucomatous brain, specifically the ventrolateral preoptic nucleus. Bang et al. also found a decreased concentration of GABA, a major inhibitory neurotransmitter, in the occipital cortex of glaucoma patients [124]. To the authors’ knowledge, this relationship has yet to be explored in glaucoma through dMRI, while proof of concept for DTI investigation of human circadian circuitry was demonstrated by Koller et al. Using dMRI tractography and FA, the researchers found an association between descending hypothalamic projections to the sympathetic nervous system and white matter microstructure in predicting daytime sleepiness [125].

dMRI was proposed as a complimentary assessment when monitoring changes associated with traumatic brain injury such as emotion, cognition, and motor control, with DTI being sensitive to the acute phase of mild traumatic brain injury [126]. A mouse study also demonstrated that repetitive mild traumatic brain injury resulted in impairments to the visual system, specifically at the retina and optic nerve [127]. The dMRI applications for monitoring microstructural changes post-concussion could be translatable to investigating comorbid conditions in glaucoma patients and determining the sensitivity of dMRI techniques to changes in white matter integrity over time.

### 4.2. Development of Clinically-Feasible Imaging Sequences

The transition from dMRI preclinical investigations to clinical applications depends on optimized imaging sequences that are within a clinically practical acquisition time. Moreover, though single-shot echo planar imaging is a common technique for diffusion dMRI data acquisition, it is susceptible to magnetic field distortions, and innovation is required in this regard. Paul et al. improved the anatomical accuracy of imaging the eye through a multi-shot diffusion-weighted Rapid Acquisition with Relaxation Enhancement (RARE) echo planar hybrid pulse sequence at 3 Tesla and 7 Tesla [128]. Their experiments demonstrated the acquisition of high-fidelity images of the eye and orbit in vivo with reduced distortion while maintaining the diffusion contrast. Future studies may integrate other techniques such as zonally magnified oblique multi-slice (ZOOM) imaging to reduce acquisition time and field of view while increasing image resolution when developing clinically feasible sequence protocols within acceptable image signal-to-noise ratios.

With recent advancements such as multiband imaging, current DKI and WMTI sequences may allow whole-brain acquisitions of 2–3 mm spatial resolution within 10 min with good image signal-to-noise ratios in clinical MRI scanners [113]. Other novel MRI techniques, such as filter-exchange imaging (FEXI), also present a promise for more specific dMRI of the molecular exchange between tissue microenvironments that can be achieved in a clinically feasible acquisition timeframe [129]. Though cylindrical axonal diameter mapping has a resolution limit between 4 and 8 µm for standard clinical MRI units, stronger gradients can attain higher resolution, and FEXI protocols may potentially serve as a starting point in seeking dMRI evidence for axonal loss in early glaucomatous states [130]. Achieving clinically optimal acquisition time would also allow larger clinical studies and clinical trials and open up opportunities for improving reproducibility of novel dMRI protocols of the visual system, ultimately helping to resolve the limited specificity from DTI measurements [129].

### 4.3. Glaucoma Detection through a Combination of Imaging Techniques

MRI allows multimodal assessments of the structural, metabolic, and functional aspects of the visual system in health and disease [131]. In order to allow investigations into different therapeutic targets in addition to intraocular pressure reduction, clinicians and researchers may opt to combine current clinical ophthalmic assessments with imaging beyond dMRI for a more comprehensive characterization of the glaucomatous visual system. For instance, while DTI had the potential for examination of tissue microstructures, magnetization transfer MRI could be more sensitive to the macromolecular environment, such as collagen and glycosaminoglycan contents in the eye and myelin contents in the white matter [132]. It is also possible that functional MRI of the visual pathway could identify the selective loss of response in the lateral geniculate nucleus and the superior colliculus of early glaucoma patients [133]. Metabolically, magnetic resonance spectroscopy may be employed to detect changes in the concentrations of neurochemicals in the glaucomatous visual pathways [97,134,135,136]. While recent studies employed dMRI techniques for the evaluation of the glymphatic brain waste clearance system by detecting intrinsic water movement along the perivascular space [137,138], dynamic contrast-enhanced MRI may also allow for more thorough identifications of changes in eye form, function, and physiology under glaucomatous conditions [12,139,140,141]. These lines of research studies may ultimately allow early glaucoma detection prior to substantial clinical vision loss or even neuronal damage onset for guiding more timely interventions [81,94].

### 4.4. Evaluation of Neurotherapeutic Options for Glaucoma

dMRI may offer the capability for the non-invasive evaluation of neuroprotective drug interventions targeting retinal ganglion cells and their axons. Based on histological data, brimonidine, an alpha-2 agonist with intraocular pressure-lowering properties, was shown to reduce the rate of retinal ganglion cell loss in rat models of optic nerve injury [142]. A recent primate study by Takahashi et al. sought to track the neuroprotective properties of brimonidine through a combination of longitudinal DTI, spectral-domain optical coherence tomography, and tonometry [143]. Their aim was to quantify the timing of intervention responsiveness in vivo within the same animals. Macaques with ocular hypertension, unilaterally induced via laser photocoagulation, were treated with either brimonidine or artificial tears as a control in both eyes twice a day. Intraocular pressure, RNFL thickness, and FA were measured at 1, 2, 3, 4, 6, and 8 weeks following laser treatments. Interestingly, albeit with relatively small sample size, the results showed that in eyes treated with brimonidine, FA did not significantly decrease over time in the optic nerve. Moreover, decreased FA was detected in the control group prior to significant RNFL thinning, possibly indicating DTI sensitivity to distal axonal degeneration prior to proximal axonal loss.

**Figure 10:**
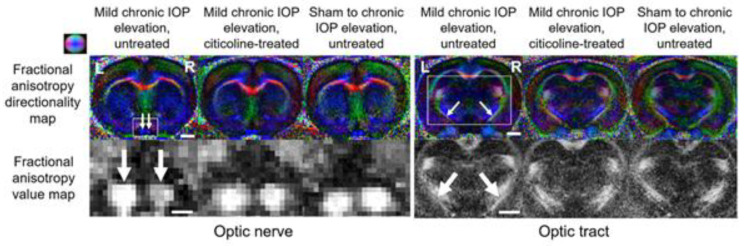
Representative DTI fractional anisotropy (FA) maps of the optic nerve (**left panel**) and optic tract (**right panel**) in an untreated rodent group with mild chronic intraocular pressure elevation to the right eye, a citicoline-treated group with mild chronic intraocular pressure elevation to the right eye, and an untreated sham group without intraocular pressure elevation. Top row illustrates the color-coded FA directionality maps, with principal diffusion directions denoted as blue (caudal-rostral), red (left-right direction), and green (dorsal-ventral). White arrows point to left and right optic nerves and optic tracts. Bottom row shows the lower FA in the right optic nerve and left optic tract of the untreated group with mild chronic intraocular pressure elevation relative to the opposite hemisphere. Such contralateral FA differences were not apparent in the other two groups. Images are adapted with permission from van der Merwe et al. [10].

Another example of longitudinal MRI evaluation of glaucoma neurotherapeutics involves oral citicoline treatment in experimental rodent models [10]. Through multiple imaging parameters, including dMRI, the researchers found less disruption of structural integrity, as indicated by reduced FA decrease and reduced RD increase in the visual pathway of the citicoline-treated group relative to the untreated group under similar levels of mild chronic intraocular elevation (Figure 10). These observations were accompanied by reduced visual acuity decrease using awake optomotor behavioral assessments, suggesting that citicoline can modulate glaucomatous neurodegeneration and visual deterioration through intraocular pressure-independent control. Successful neuroprotection is dependent on early disease detection, biomarker identification, and multiple pathway targets [144], all of which have proven challenging in glaucoma research. Nevertheless, dMRI shows promise as a tool to shed light on these issues. Future clinical trials are envisioned that use dMRI and other imaging modalities to examine not only optic nerve degeneration but also trans-neuronal degeneration in glaucoma patients [28].

## 5. Conclusions

Diffusion MRI of the visual pathway, from the eye to the visual cortex and beyond, may contribute to key evidence characterizing glaucoma as a trans-synaptic disease involving both the brain and the eye, and may inform the neurodegenerative mechanisms that implicate the disorder. Due to the level of complexity of the biological system as well as the glaucoma pathogenesis, further basic and translational investigations are needed to improve this imaging modality and the analysis models, and to relate imaging results to the underlying biological processes better. Despite existing technical and clinical challenges, the inherent flexibility of MRI sequences supports the development of novel dMRI methods for improving the specificity of tissue microstructure characterization for a more comprehensive description of glaucomatous changes in humans and experimental animal models. DTI offers a key and sensitive tool for detecting and investigating white matter changes in the glaucomatous brain in vivo, while the higher-order dMRI models may lead to more robust gray matter characterization and better interpretation of the biological significance of the DTI findings in the glaucoma literature. The increasing availability of dMRI data may also help develop better pathological profiles and to determine the similarities and differences for diseases affecting the eye and visual pathways. Overall, advanced dMRI of the glaucomatous visual system may play an important role in identifying novel biomarkers and neurotherapeutic targets, as well as in promoting early detection, timely intervention, and outcome monitoring so as to reduce the prevalence of this irreversible but preventable disease.

## Figures and Tables

**Table 1:** Equations for deriving DTI parameters from the eigenvalues (*λi*) in each imaging voxel. *i* = 1 represents the principal diffusion direction, while *i* = 2 and 3 correspond to the orthogonal diffusion directions perpendicular to the principal diffusion direction.

DTI Parameter	Equation	
Mean Diffusivity (MD)	(*λ*_1_ + *λ*_2_ + *λ*_3_)/3	(2)
Fractional Anisotropy (FA)	√(3[(*λ*_1_ − MD)^2^ + (*λ*_2_ − MD)^2^ + (*λ*_3_ − MD)^2^])/(2(*λ*_1_^2^ + *λ*_2_^2^ + *λ*_3_^2^)]	(3)
Axial Diffusivity (AD)	*λ*_1_	(4)
Radial Diffusivity (RD)	(*λ*_2_ + *λ*_3_)/2	(5)

**Table 2:** Summary of DTI parametric changes in the optic nerve of glaucoma patients relative to healthy controls in selected articles.

Glaucoma Type	DTI Parametric Change				Field Strength (T)	Reference
	FA	AD	RD	MD		
Normal-Tension Glaucoma	↓	↑	↑	↑	1.5	[31]
Normal-Tension Glaucoma	↓	↑	NA	NA	1.5	[77]
Normal-Tension Glaucoma	↓	NA	NA	NA	7	[87]
Normal-Tension Glaucoma	↓	↓	↑	↑	3	[81]
Normal-Tension Glaucoma	↓	NS	↑	NA	3	[88]
Primary Open-Angle Glaucoma	↓	NA	NA	↑	3	[89]
Primary Open-Angle Glaucoma	↓	NS	↑	NS	3	[90]
Primary Open-Angle Glaucoma	↓	NS	↑	NA	3	[88]
Primary Open-Angle Glaucoma	↓	↑	↑	↑	3	[83]
Primary Open-Angle Glaucoma	↓	NA	NA	NA	3	[85]
Primary Open-Angle Glaucoma	↓	NA	NA	↑	3	[91]
Primary Open-Angle Glaucoma	↓	NA	NA	↑	3	[92]

↓ = DTI parameter being lower in glaucoma than healthy control subjects; ↑ = DTI parameter being higher in glaucoma than healthy control subjects; NS = non-significant result; NA = result not available.

**Table 3:** Summary of DTI parametric changes across visual brain regions of glaucoma patients relative to healthy control subjects in selected articles. LGN: Lateral geniculate nucleus; V1: Primary visual cortex; NTG: Normal-tension glaucoma; POAG: Primary open-angle glaucoma.

	Optic Nerve	Optic Tract	Optic Radiation (LGN to V1)	LGN	Field Strength (T)	Glaucoma Type	Reference
Fractional Anisotropy	↓	↓	↓	NA	7	NTG	[87]
Fractional Anisotropy	↓	NA	↓	NA	3	POAG	[72]
Fractional Anisotropy	NA	NS	↓	NS	3	POAG	[92]
Radial Diffusivity	↑	NA	↑	NA	3	POAG	[72]
Mean Diffusivity	NA	↑ *	NS	↑	3	POAG	[92]

↓ = DTI parameter being lower in glaucoma than healthy control subjects; ↑ = DTI parameter being higher in glaucoma than healthy control subjects; * Significance observed on left side only; NS = non-significant result; NA = result not available..
